# *In Vivo* Tumor Growth Rate Measured by US in Preoperative Period and Long Term Disease Outcome in Breast Cancer Patients

**DOI:** 10.1371/journal.pone.0144144

**Published:** 2015-12-10

**Authors:** Tae-Kyung Yoo, Jun Won Min, Min Kyoon Kim, Eunshin Lee, Jongjin Kim, Han-Byoel Lee, Young Joon Kang, Yun-Gyoung Kim, Hyeong-Gon Moon, Woo Kyung Moon, Nariya Cho, Dong-Young Noh, Wonshik Han

**Affiliations:** 1 Department of Surgery, Seoul National University College of Medicine, Seoul, Korea; 2 Department of Surgery, Dankook University College of Medicine, Cheonan, Korea; 3 Department of Radiology, Seoul National University College of Medicine, Seoul, Korea; 4 Laboratory of Breast Cancer Biology, Cancer Research Institute, Seoul National University College of Medicine, Seoul, Korea; University of North Carolina School of Medicine, UNITED STATES

## Abstract

**Objective:**

The aim of our study was to evaluate the effect of tumor growth rate, calculated from tumor size measurements by US, on breast cancer patients’ outcome.

**Patients and Methods:**

Breast cancer patients who received at least two serial breast ultrasonographies (US) in our institution during preoperative period and were surgically treated between 2002 and 2010 were reviewed. Tumor growth rate was determined by specific growth rate (SGR) using the two time point tumor sizes by US.

**Results:**

A total of 957 patients were analyzed. The median duration between initial and second US was 28 days (range, 8–140). The median initial tumor size was 1.7cm (range, 0.4–7.0) and median second size was 1.9cm (range, 0.3–7.2). 523(54.6%) cases had increase in size. The median SGR(x10^-2^) was 0.59 (range, -11.90~31.49) and mean tumor doubling time was 14.51 days. Tumor growth rate was higher when initial tumor size was smaller. Lymphovascular invasion, axillary lymph node metastasis, and higher histologic grade were significantly associated with higher SGR. SGR was significantly associated with disease-free survival (DFS) in a univariate analysis (p = 0.04), but not in a multivariate Cox analysis (p>0.05). High SGR was significantly associated with worse DFS in a subgroup of initial tumor size >2cm (p = 0.018), but not in those with tumor size <2cm (p>0.05).

**Conclusion:**

Our results showed that tumor growth rate measured by US in a relatively short time interval was associated with other worse prognostic factors and DFS, but it was not an independent prognostic factor in breast cancer patients.

## Introduction

Tumor growth rate has always been a matter of interest, not only as a quantifiable character of the tumor but also as a tool to plan and evaluate screening programs, clinical trials or epidemiologic studies. Most studies have used data obtained from screening mammographies, determining tumor growth rate by using biomathematical estimations with various growth patterns [1–4]. Also under the assumption that rapidly growing tumors present with aggressive features, the prognostic significance of tumor growth rate in breast cancer has been evaluated in several retrospective studies, mostly presenting with inverse association between patient survival and tumor growth rate [5–8]. However tumor growth rate has not been used as a prognostic variable in clinical practice, due to the difficulty of evaluating it in the short interval between diagnosis and treatment.

In previous studies, mammography has been one of the main tools in evaluating tumor growth rate. However, mammography is not a reliable tool to measure tumor size, especially in dense breasts and small tumors [9, 10]. Also considering the high percentage of dense breast in Asian women [11, 12], mammography is insufficient for serial tumor size measurement. In comparison, breast ultrasonography(US) is more accurate in measuring tumor size in dense breasts, and also repetitive evaluation is feasible due to its nonionizing method(10). Furthermore, considering its accuracy, breast US can assess minimal tumor size changes presented in a short interval.

The most commonly used tumor growth model is exponential growth and Gompertz growth [1, 4]. In short intervals exponential growth is commonly used, as Gompertz growth cannot be calculated due to the lack of information for estimating the needed parameters [13, 14]. Exponential growth is generally quantified as doubling time(DT). But Mehrara et al. [15] pointed out that the mean value of DT does not indicate the average growth rate and is not suitable for statistical testing. Under this perception, Mehrara et al. proposed an alternative method of quantifying growth rate, specific growth rate(SGR), calculated to be equal to ln2/DT. Compared to DT, SGR has been shown to be more suitable for short measurement time intervals, be least influenced by uncertainties of measurement procedure and uniformly reflects the difference between growth rates throughout all ranges [16].

In this retrospective study, we determined breast cancer tumor growth rate, expressed as SGR, by measurement of tumor size at two time points before treatment, via US performed at a single institution. The objective of this study was to investigate the relationship between breast cancer growth rate and clinicopathologic factors and patient survival.

## Patients and Methods

### Patients and clinicopathologic data

Patients who received surgery for primary invasive breast cancer at Seoul National University Hospital (SNUH) between January 2002 and December 2010 were retrospectively reviewed. Patients who received at least two serial breast US in SNUH at initial visit (1^st^ US) and at one day before surgery (2^nd^ US) with an interval of more than 7 days were included in the study. Patients who had a difference of over 1cm between 2^nd^ US tumor size and pathologic tumor size were excluded, premising that in these cases preoperative US evaluation did not demonstrate the invasive portion of the tumor correctly. US units equipped with 10- or 12-MHz linear-array transducers (LOGIQ 700 scanner, GE Healthcare; iU22, Philips Healthcare) were used for the procedures and was performed by radiologists with 2–25 years of experience in breast US. Clinicopathological data were obtained from SNUH Breast Cancer Center database [17], which is a prospectively maintained web-based database. US readings were retrospectively reviewed via electronic medical records, to acquire measured maximal diameter of the tumor. Recurrence event data was collected from review of electronic medical records and survival data was obtained from the Korean National Statistical Office database.

### Specific Growth Rate (SGR)

SGR was calculated by the following equation: [15]

$$\text{V}2=\text{V}1\times e^{SGR\times (T-To)}$$

and

$$\text{SGR}=\text{ln}(\frac{V}{Vo})/(T-To)$$

and

$$\text{SGR}=3\times \text{ln}(\frac{D}{Do})/(T-To)$$

(T-T_0_) indicate the time interval between 1^st^ and 2^nd^ US.

D_0_ and D indicate maximal tumor diameters at 1^st^ and 2^nd^ US, respectively.

### Statistical Analysis

Univariate analysis was performed to analyze the relationship between SGR and clinicopathologic features of breast cancer. Pearson chi-square test for categorical variables and Student’s t-test and analysis of variance (ANOVA) were used to compare continuous variables. Univariate survival analysis to compare survival between different tumor growth rates was done using Kaplan-Meier survival analysis and log-rank tests. Multivariate survival analysis was conducted using Cox proportional hazards regression model, adjusting for factors that differ between different tumor growth rate groups or factors that are known to influence survival. Primary endpoint of this study was DFS. Disease-free survival (DFS) was defined as the time from surgery to the date of either breast cancer recurrence, death from any cause or final outpatient clinic visit. Breast cancer recurrence only included first locoregional recurrence or distant metastasis.

This study was approved by the Institutional Review Board of Seoul National University Hospital and the committee waived for informed consent (IRB no. 1508-167-699). All investigations have been conducted according to the principles expressed in the Declaration of Helsinki and all patient records and information were anonymized and de-identified prior to analysis.

## Results

We identified 1,257 patients who received serial breast US examinations at SNUH preoperatively between 2002 and 2010. Of these, 300 patients were excluded due to short examination interval or large difference between 2^nd^ US tumor size and pathologic tumor size. A final 957 patients were included for analysis.

The median age at diagnosis was 50 years old (range, 22–83 years). The median duration between 1^st^ and 2^nd^ US was 28 days (range, 8–140 days) and median interval between diagnostic biopsy and surgery was 26 days (range, 1–157 days). The median D_0_ was 1.7cm (range, 0.4–7.0cm), median D was 1.9cm (range, 0.3–7.2cm) and the median difference between D and D_0_ was 0.1cm (range, -2.0–4.1cm). 198 (20.7%) patients had a smaller tumor size, 236 (24.7%) patients had the same tumor size and 523 (54.6%) had a larger tumor size at 2^nd^ US compared to 1^st^ US. The median size increase rate ((D-D_0_)/D_0_) was 5.88% (range, -166.67–73.17%) and median SGR(x10^-2^) was 0.59 (range, -11.90–31.49) with mean SGR(x10^-2^) of 1.12±3.02. Tumor doubling time (DT, ln/SGR) ranged from -565.92 days to 387.55 days with mean DT of 14.51 days.

The patient and tumor characteristics and its correlation with tumor SGR is shown in Table 1. At t-test analysis, smaller 1^st^ US size and lymphovascular invasion were associated with higher SGR. When SGR was dichotomized by median value, axillary lymph node metastasis and higher histologic grade were also related to higher SGR. Although sample size was small, very young patients (<30yrs) presented with an almost 3 times faster SGR. SGR did not differ between tumor subtypes.

**Table 1:** Correlation between clinicopathologic features and SGR.

		Result, n = 958	Mean SGR^1^(x10^-2^)	p-value^2^	Low SGR^1^ (x10^-2^) (<0.59)	High SGR^1^ (x10^-2^) (>0.59)	p-value^3^
**Age at diagnosis**	≤30	11	3.26	0.272	3 (0.6)	8 (1.7)	0.626
	31–40	129	1.04		69 (14.4)	60 (12.6)	
	41–50	349	1.00		178 (37.2)	171 (35.8)	
	51–60	282	1.21		138 (28.8)	144 (30.1)	
	61–70	157	1.14		78 (16.3)	79 (16.5)	
	71≤	29	1.09		13 (2.7)	16 (3.3)	
**US interval (days)**	8–18	232	1.41	0.069	127 (26.5)	105 (22.0)	0.333
	19–28	277	1.32		131 (27.3)	146 (30.5)	
	29–40	218	0.78		111 (23.2)	107 (22.4)	
	41 -	230	0.91		110 (23.0)	120 (25.1)	
**1** ^**st**^ **US size (cm)**	≤ 1	170	2.10	< 0.001	77 (16.1)	93 (19.5)	0.104
	>1, ≤ 1.5	258	1.34		119 (24.8)	139 (29.1)	
	>1.5, ≤ 2	217	0.98		112 (23.4)	105 (22.0)	
	> 2	312	0.50		171 (35.1)	141 (29.5)	
**Axillary Lymph Node status**	Negative	621	1.02	0.170	332 (69.3)	289 (60.5)	0.004
	Positive	336	1.30		147 (30.7)	189 (39.5)	
**Tumor Grade (n = 895)**	Grade 1,2	478	1.09	0.735	263 (58.4)	215 (48.3)	0.002
	Grade 3	417	1.16		187 (41.6)	230 (51.7)	
**Lymphovascular Invasion (n = 884)**	Negative	550	0.85	0.006	298 (67.3)	252 (57.1)	0.002
	Positive	334	1.40		145 (32.7)	189 (42.9)	
**Ki-67 (n = 941)**	< 10%	733	1.09	0.650	377 (80.0)	356 (75.7)	0.112
	≥ 10%	208	1.19		94 (20.0)	114 (24.3)	
**Hormone Receptor**	Positive	697	1.18	0.330	354 (73.9)	343 (71.8)	0.455
	Negative	260	0.96		125 (26.1)	135 (28.2)	
**Subtype (n = 952)**	Luminal	697	1.18	0.627	354 (74.4)	343 (72.1)	0.569
	HER2	67	0.99		34 (7.1)	32 (6.7)	
	TNBC	189	0.95		88 (18.5)	101 (21.2)	

^1^ SGR Specific Growth Rate, US Ultrasonography

^2^ *p* values are from T-test & ANOVA test

^3^ *p* values are from χ2 test

**Fig 1:**
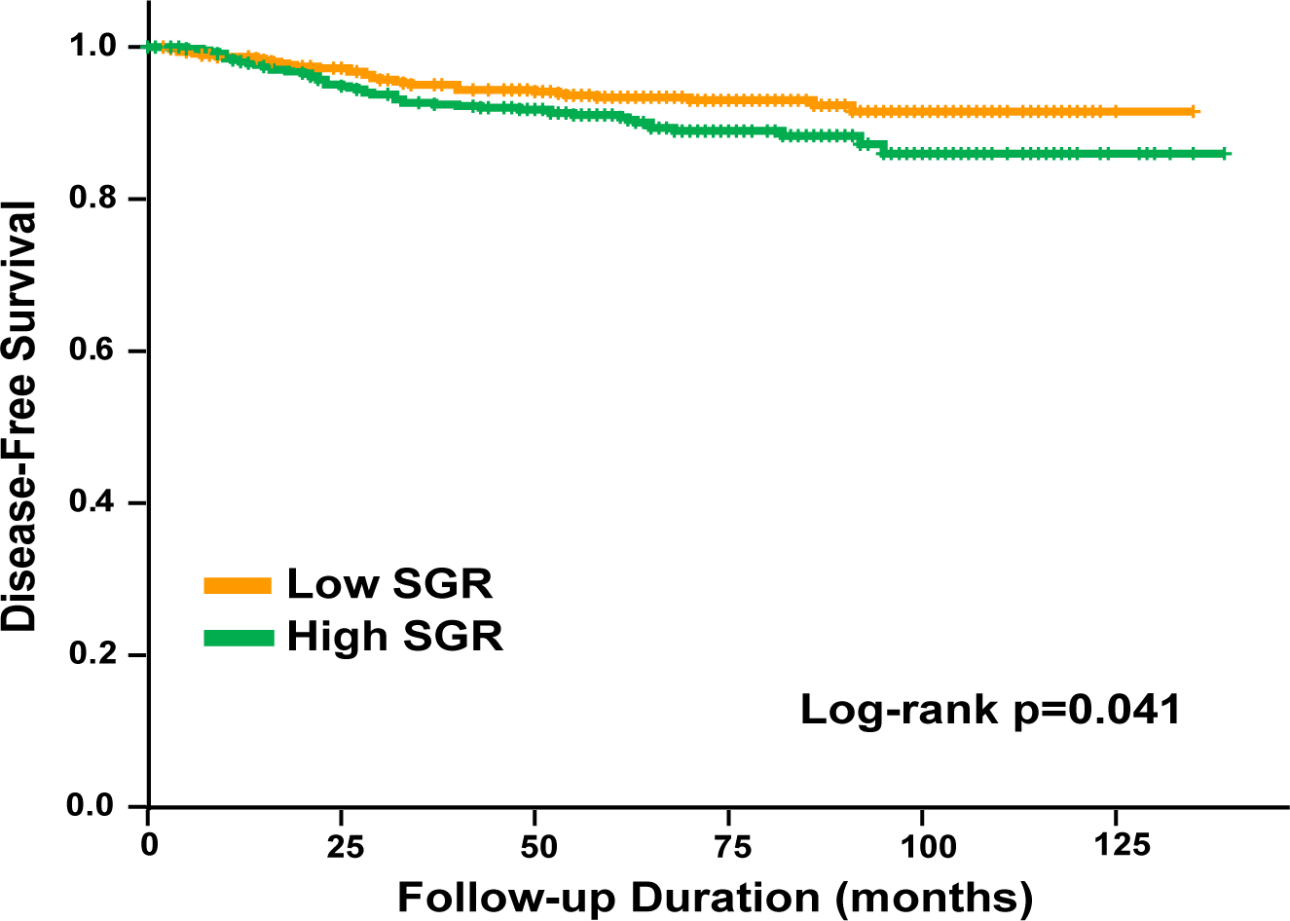
DFS according to low and high SGR (log rank test p = 0.041).

The median follow-up period of the patients was 70.0 months (range, 0–139 months). 5-year overall survival (OS) and 5-year disease-free survival (DFS) rates were 96.5% and 92.2%, respectively. Patients were dichotomized by use of median SGR as a cutoff value (<0.59x10^-2^ vs ≥0.59x10^-2^). There was a significant difference in DFS between low and high SGR groups (log rank test p = 0.041, Fig 1). In a univariate analysis, SGR was an independent risk factor for DFS, however, when adjusted for other known prognostic factors, SGR did not remain a significant predictor of DFS (HR = 1.175; 95% CI, 0.754 to 1.868, p = 0.489, Table 2).

**Table 2:** Cox regression analysis for DFS.

		Univariate			Multivariate		
		HR^1^	95% CI^2^	p value	HR	95% CI	p value
**T size**	continuous	1.631	1.414, 1.881	<0.001	1.388	1.159, 1.664	<0.001
**LN status**	(+) vs. (-)	2.642	1.709, 4.085	<0.001	1.711	1.028, 2.847	0.039
**Tumor Grade**	Gr 3 vs. Gr 1,2	5.188	3.004, 8.961	<0.001	3.357	1.801, 6.258	<0.001
**Lymphovascular Invasion**	Yes vs. No	2.370	1.527, 3.678	<0.001	1.130	0.683, 1.870	0.634
**Ki67**	≥10% vs <10%	1.762	1.113, 2.790	0.016	0.812	0.488, 1.351	0.423
**ER and/or PR**	(-) vs. (+)	2.533	1.644, 3.901	<0.001	1.432	0.861, 2.383	0.166
**SGR (x10** ^**-2**^ **)**	High vs. Low	1.574	1.014, 2.443	0.043	1.175	0.754, 1.853	0.489

^1^HR Hazard Ratio obtained by Cox proportional hazard models

^2^CI Confidence Interval

**Fig 2:**
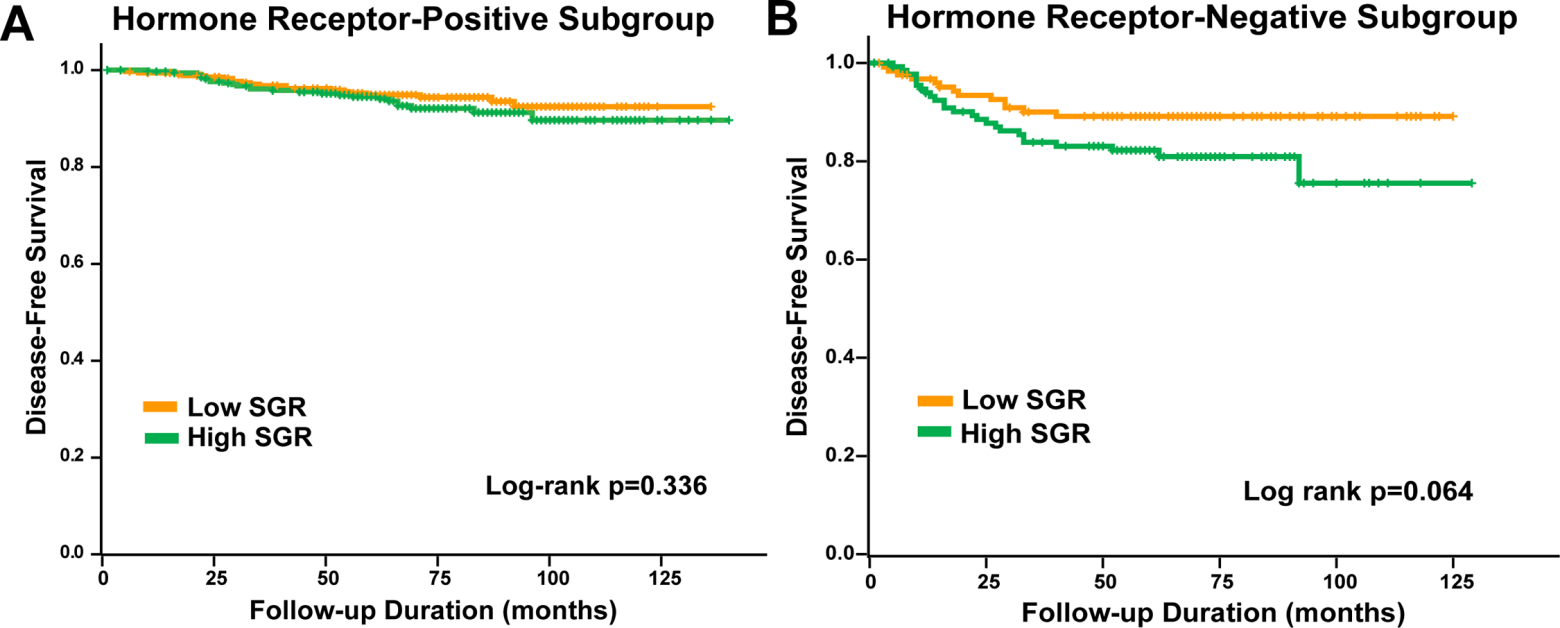
DFS by hormone-receptor status. (A) DFS according to low and high SGR in hormone receptor-positive subgroup (log rank test p = 0.336). (B) DFS according to low and high SGR in hormone receptor-negative subgroup (log rank test p = 0.064).

**Fig 3:**
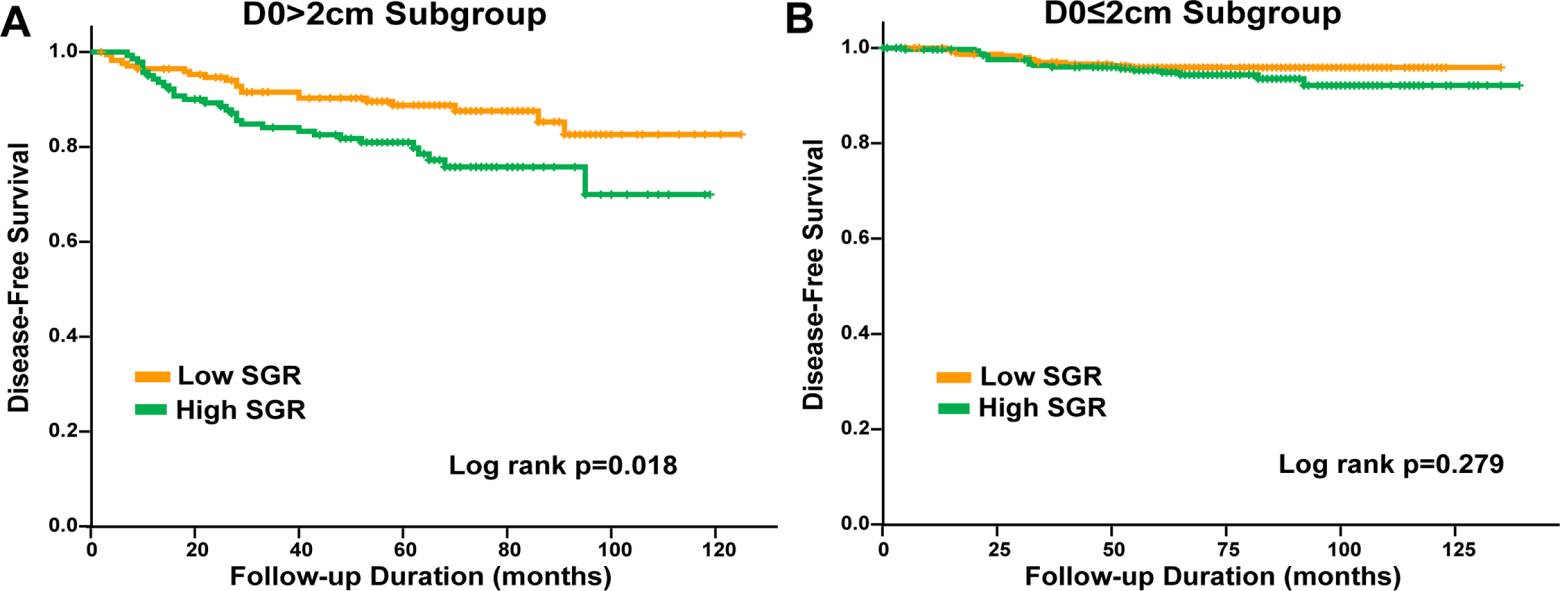
DFS by initial tumor size (D_0_). (A) DFS according to low and high SGR in D_0_≤2cm subgroup (log rank test p = 0.277). (B) DFS according to low and high SGR in D_0_>2cm subgroup (log rank test p = 0.018).

Subgroup analysis for survival was done, by hormone receptor status and initial tumor size (D_0_) (D_0_ ≤ 2cm, D_0_ > 2cm). DFS did not significantly differ according to SGR in the hormone receptor-positive (log rank test p = 0.336, Fig 2A) and hormone receptor-negative subgroup (p = 0.064, Fig 2B). Patients with high SGR showed significantly low DFS in the D_0_>2cm subgroup (log rank test p = 0.018, Fig 3A and 3B), but not in the D_0_≤2cm subgroup. In a multivariate analysis in D_0_>2cm subgroup, high SGR was not a significant independent factor for worse DFS (HR 1.708; 95% CI 0.938 to 3.111, p = 0.080, Table 3).

**Table 3:** Cox regression analysis for DFS in D_0_>2cm.

		Univariate			Multivariate		
		HR^1^	95% CI^2^	p value	HR^1^	95% CI^2^	p value
**T size**	continuous	1.221	0.976, 1.527	0.080	1.126	0.859, 1.476	0.390
**LN status**	(+) vs. (-)	1.663	0.951, 2.908	0.074	1.230	0.650, 2.327	0.524
**Tumor Grade**	Gr 3 vs. Gr 1,2	2.345	1.141, 4.820	0.020	1.841	0.829, 4.089	0.134
**Lymphovascular Invasion**	Yes vs. No	1.754	0.990, 3.106	0.054	1.226	0.643, 2.337	0.536
**Ki67**	≥10% vs <10%	1.340	0.766, 2.346	0.305	0.866	0.466, 1.609	0.650
**ER and/or PR**	(-) vs (+)	1.753	1.017, 3.020	0.043	1.376	0.737, 2.571	0.317
**SGR (x10** ^**-2**^ **)**	High vs. Low	1.933	1.109, 3.367	0.020	1.708	0.938, 3.111	0.080

^1^HR Hazard Ratio obtained by Cox proportional hazard models

^2^CI Confidence Interval

## Discussion

Breast cancer is a heterogeneous disease, not only presenting with widely different prognosis, but also known to have a wide range of tumor growth rate [14, 18]. In this study, we determined the tumor growth rate in breast cancer using sequential tumor size measurement done by US in a short interval and analyzed whether it is associated with other characteristics of the tumor and patients, and further, whether it is associated with long term outcome of the patients. As a result, the tumor growth rate was correlated with some aggressive tumor characteristics and disease-free survival in univariate analysis, but not in multivariate analysis. This study, to our knowledge, is the first one that observed the tumor size change *in vivo* using US and analyzed its relation to survival outcome.

Previous literatures have addressed that faster growing tumors are associated with worse survival, but these studies determined tumor growth rate indirectly or using inconstant methods [6–8]. Galante et al. applied tumor size difference using two mammographic examinations and reported that tumor growth rates do not influence disease-free probabilities, but are of prognostic value when the disease is not localized [5]. In contrary, our findings demonstrate that tumor growth rate has no association to patient survival, especially in early breast cancer. However, direct comparison of these reports to our study is not suitable, as tumor growth rate estimation methods differ and shorter interval was applied in this study. Also all previous reports are from over 20 years ago, not reflecting the dramatic change in breast cancer treatment during the recent decades.

Interval between diagnosis and treatment initiates anxiety in patients and their family and is an important component related to health care quality [19]. Our center has recently reported that delay of treatment initiation in early breast cancer does not adversely affect survival outcome(unpublished data). Wagner et al. did not proceed with survival analysis but did report that modest treatment delays are not significantly associated with change in tumor size [20]. Although with different approaches, these reports are consistent with our results, suggesting that patients, especially patients with early breast cancer, may undergo preoperative evaluation without concern of disease progression.

In this study, quantified tumor growth rate was relatively higher compared to previous literatures. Mean DT was 14.51 days in our study, whereas previously reported mean or median DT ranged from 60 days to 270 days [2, 7, 13, 14, 18, 21, 22]. Previous studies that were based on inpatients using serial measurements have presented with relatively faster growing tumors [14]. However, even considering this difference in study method, mean DT in our study was relatively short. In our study, most of the cases performed intervention procedures, mostly gun biopsies, between the two USs, which might have affected the US tumor size measurement. Also, relatively short interval between tumor size measurements in our study would have influenced the results. In spite of this difference from previous literatures, our approach of using an interval between diagnosis and treatment is practical, enabling application of our results directly into the clinic.

Subgroup analysis demonstrated that when a tumor has an initial size of over 2cm, high tumor growth rate presents with a tendency for poor DFS. Growth of small tumors is mostly regulated by cell reproduction rate, presenting with an exponential growth curve with constant doubling times. As for larger tumors, growth rate normally decreases as the tumor receives limited nutrition [4]. Our study suggests that despite this reduced growth rate, when a large tumor presents with high tumor growth rate, the tumor is likely to present with aggressive features, resulting with poor patient survival.

In previous studies, high cell proliferation markers are usually correlated with worse outcome in hormone receptor-positive tumors [23, 24]. However, in our study, SGR was not associated with DFS in hormone receptor-positive tumors. Imaging findings are known to differ among tumor phenotype in breast cancer. Luminal cancers are more likely to show irregular shapes with irregular or speculated margins. On the other hand, triple negative or human epidermal growth factor receptor 2(HER2)-overexpression cancers frequently present with round or oval shapes with smooth margins [25, 26]. Due to its irregular shape, diameter change in hormone responsive cancers may not accurately reflect tumor growth rate, compared to non-hormone responsive cancers. As a result, in hormone responsive cancers, tumor growth rate difference measured by serial US may not accurately reflect actual diameter difference, resulting with no influence on patient survival. No previous studies have presented the prognostic value of tumor growth rate according to tumor size or subtype.

Factors associated with tumor growth rate present with similar results to previous reports. Larger initial tumor size was related to lower tumor growth rate, demonstrating parabolic growth or Gompertz growth, as previous studies have shown [1, 3]. Also higher growth rate was associated with axillary lymph node metastasis, lymphovascular invasion, and histologic grade. Similar results have been reported before [5, 6, 13, 22], demonstrating the simple assumption of rapidly growing tumors presenting with aggressive feature. Unexpectedly, Ki-67 level, a surrogate marker of cell proliferation rate, did not correlate with SGR. Many papers have reported that breast cancer grows faster in young age patients [2, 13, 14, 22]. Although our study showed no significant difference among age groups, very young patients(≤30 years old) presented with a mean SGR almost 3 times higher than other age groups.

The current study has several limitations. First, the interval between sequential breast USs was relatively short and intervention to the tumor was performed in most of the cases. As a result, almost half of the cases had smaller or same size tumors at second US. This is also related to the lack of objectivity of US examination itself. To overcome the uncertainties of measurement procedures, SGR was used to measure tumor growth rate [15] and only cases that received breast US at our institution by experienced radiologists were included. Second, tumor volumes were calculated using only one dimension, assuming that the tumor shape is a sphere. Breast tumors have been observed to grow to extend in one axis forming an oblate spheroid or a cylinder [21]. Measuring tumor size using MRI images will allow more objective measurement and precise calculation of tumor growth rate. However repetitive MRI studies are not feasible considering its cost and inconvenience during examination. Third, as we excluded patients who received neoadjuvant chemotherapy, patients with more aggressive tumors might have been excluded selectively.

In conclusion, our results showed that *in vivo* tumor growth rate measured by US in a relatively short time interval was associated with other worse prognostic factors and DFS, but it was not an independent prognostic factor in breast cancer patients. Future studies with larger sample size and more objective measure such as serial breast MRI will be able to provide a more definite answer.
